# Di-*tert*-butyl­tin(IV)–hydroxide–iodide, ^*t*^Bu_2_Sn(OH)I, the last missing member in the series of pure di-*tert*-butyl­tin(IV)–hydroxide–halides

**DOI:** 10.1107/S205698902200514X

**Published:** 2022-05-20

**Authors:** Hans Reuter

**Affiliations:** a Chemistry, Osnabrück University, Barbarastr. 7, 49069 Osnabrück, Germany; Vienna University of Technology, Austria

**Keywords:** crystal structure, hydrolysis, trigonal-bipyramidal coordination, van-der-Waals inter­action

## Abstract

The crystal structure of di-*tert*-butyl­hydroxido­iodido­tin(IV), [Sn(C_4_H_9_)_2_I(OH)] or ^*t*^Bu_2_Sn(OH)I, consists of dimeric, centrosymmetric mol­ecules exhibiting the typical structural features of diorganotin(IV)-hydroxide-halides, *R*_2_Sn(OH)*Hal*. Two trigonal–bipyramidally coordinated tin(IV) atoms are bridged *via* two hydroxyl groups, resulting in a planar, four-membered {Sn—O}_2_ ring of rhombic shape, with acute angles at tin, obtuse angles at oxygen and two different Sn—O distances depending whether the oxygen atom adopts an axial or equatorial position at the tin(IV) atom. In contrast to its fluorine, chlorine and bromine homologues, no hydrogen bonds between the OH group and the halide atom exist, confining the inter­molecular inter­actions to van der Waals forces.

## Abstract 2

The crystal structure of di-*tert*-butyl­hydroxido­iodido­tin(IV), [Sn(C_4_H_9_)_2_I(OH)] or ^*t*^Bu_2_Sn(OH)I, consists of centrosymmetric dimers exhibiting the characteristic structural features of diorganotin(IV)-hydroxide-halides.

## 1. Chemical context

During the hydrolysis of diorganotin(IV)-dihalides, *R*_2_Sn*Hal*_2_, to diorganotin(IV) oxides, *R*_2_SnO, various inter­mediates are formed. The most prominent ones are the diorganotin-hydroxide-halides, *R*_2_Sn(OH)*Hal*, the tetra­organo-dihalogenide-distannoxanes, (*R*_2_Sn*Hal*)_2_O, the tetra­organo-hydroxide-halide-distannoxanes, (*R*_2_Sn*Hal*)O(*R*_2_SnOH), and the tetra­organo-di­hydroxide-distannoxanes, (*R*_2_SnOH)_2_O. In solution as well as in the crystalline state, all these compounds are comprised of dimeric mol­ecules, and their compositions and structures result from a sequence of different hydrolysis, aggregation and condensation reactions.

In all three different classes of tetra­organo-distannoxanes, numerous compounds have been structurally characterized. All of therm exhibit a *ladder*-type arrangement of the Sn—O—*Hal*/OH framework [e.g. *dihalogenides*: *R* = Ph, Hal = Cl (Vollano *et al.*, 1984 ▸), *R* = ^*i*^Pr, *Hal* = Br (Beckmann *et al.*, 2002*a* ▸); *hydroxide-halides*: *R* = Et, *Hal* = Cl (Momeni *et al.*, 2019 ▸), *R* = Ph, *Hal* = Br (Yap *et al.*, 2010 ▸); *di­hydroxides*: *R* = neophyl (Reuter & Pawlak, 2000 ▸), *R* = tri­methyl­silylmethyl (Beckmann *et al.*, 2002*b* ▸)].

Pure *hydroxide halides* have been prepared and structurally characterized for ^*t*^Bu_2_Sn(OH)*Hal* with *Hal* = F, Br (Puff *et al.*, 1985 ▸), *Hal* = Cl (*α*-modification: Puff *et al.*, 1985 ▸; *β*-modification: Di Nicola *et al.*, 2011 ▸) and for *R* = *p*-tolyl and *Hal* = Br (Lo & Ng, 2009 ▸). Their structures are dominated by various –OH⋯*Hal* bridges between neighbouring mol­ecules, resulting in their chain-like arrangements. *Hydroxide halides* can be isolated when their hydroxyl groups are involved in hydrogen bonds to Brønstedt bases (*BB*). Such adducts of formula [*R*_2_Sn(OH)*Hal*]·2*BB* have been described for *R* = Ph, *Hal* = Cl with *BB* = EtOH (Barba *et al.*, 2007 ▸), and *BB* = quinoline (Anacona *et al.*, 2003 ▸). 
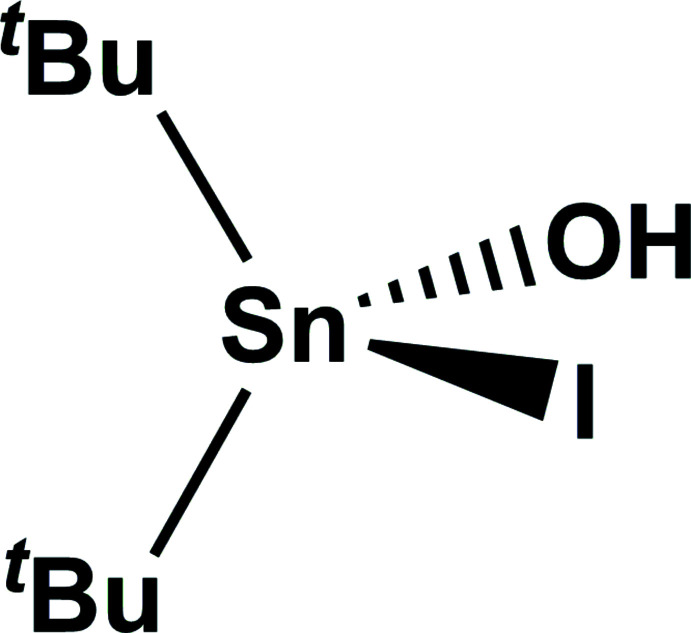


Here we present the mol­ecular and crystal structure of the last missing member in the series of pure di-*tert*-butyl­tin hydroxide halides where *Hal* = I. The analogous mol­ecule with DMSO as a hydrogen-bonded Brønsted base was formerly found as part of co-crystals with [(^*t*^Bu_2_Sn)_3_O(OH)_2_I]I (Reuter & Wilberts, 2014 ▸).

## 2. Structural commentary

**Figure 1:**
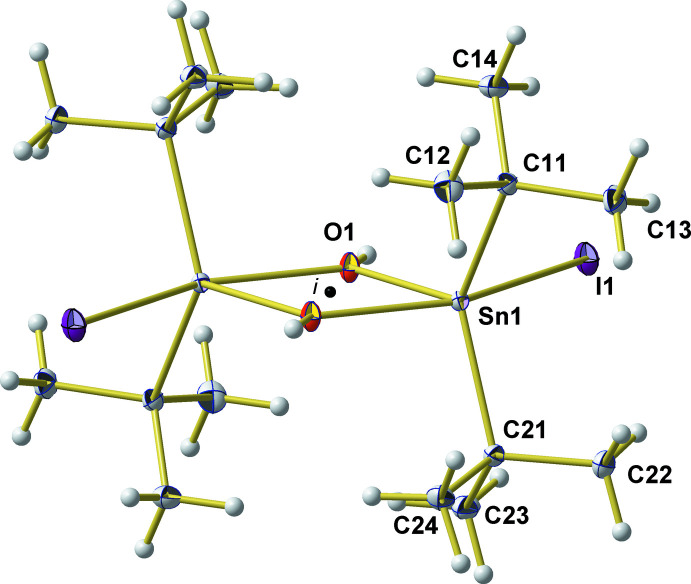
Ball-and-stick model of the dimeric, centrosymmetric mol­ecule found in the crystal of ^*t*^Bu_2_Sn(OH)I, with atom numbering of the asymmetric unit. With the exception of the hydrogen atoms that are shown as spheres of arbitrary radius, all other atoms are drawn as displacement ellipsoids at the 40% probability level. The black dot labelled *i* indicates the position of the centre of symmetry.

The asymmetric unit of the title compound comprises one ^*t*^Bu_2_Sn(OH)I moiety that dimerizes to form a centrosymmetric mol­ecule (Fig. 1 ▸)*.* As in all other *hydroxide halides*, dimerization occurs *via* the two hydroxyl groups that act as bridges between two trigonal–bipyramidally (tbpy) coordin­ated Sn^IV^ atoms.

The anisotropic displacement parameters as well as the small isotropic displacement parameters of the hydrogen atoms (see *Refinement*) indicate a negligibly small rotation of the *tert*-butyl groups as a whole and a small rotation of the methyl groups in particular, giving rise to very precise information on bond lengths and angles. The structural features of the *tert*-butyl groups are characterized by C—C bond lengths in the range 1.524 (3) to 1.533 (3) Å [mean value: 1.529 (3) Å], C—C—C angles in the range 109.5 (2) to 111.3 (2)° [mean value: 110.2 (7)°], Sn—C bond lengths between 2.187 (2) and 2.193 (2) Å [mean value: 2.190 (3) Å], and Sn—C—C angles of 107.8 (1)° to 109.6 (1)° [mean value: 108.8 (9)°]. All these values are more precise in comparison with those of the formerly determined di-*tert*-butyl­tin hydroxide halides (Puff *et al.*, 1985 ▸; Di Nicola *et al.*, 2011 ▸), especially as a result of low-temperature measurement and high data redundancy combined with multi-scan absorption correction, but are of the same accuracy and absolute value as those of the DMSO adduct [(^*t*^Bu_2_Sn)_3_O(OH)_2_I]I [*d*(C—C) = 1.529 (4) Å, 〈(C—C—C) = 109.9 (4)°, *d*(Sn—C) = 2.193 (10), 〈(Sn—C—C) = 109.4 (7)°; Reuter & Wilberts, 2014 ▸]. These data are confirmed by a redetermination of the crystal structure of the *α*-modification of [^*t*^Bu_2_Sn(OH)Cl)]_2_ (Reuter, 2022 ▸) performed with similar experimental conditions as for the title compound and its co-crystallizate. In this context, Sn—C distances are of special inter­est as they belong to the longest ones observed in case of Sn in a trigonal–bipyramidal coordination. In the other hydroxide halides mentioned above, the following bond lengths have been found: *d*(Sn—C)_mean_ = 2.120 (8) Å for *R* = *p*-tolyl, *Hal* = Br; *d*(Sn—C)_mean_ = 2.121 (10)/2.117 (4) Å for *R* = Ph, *Hal* = *Cl*, *BB* = quinoline/EtOH.

**Figure 2:**
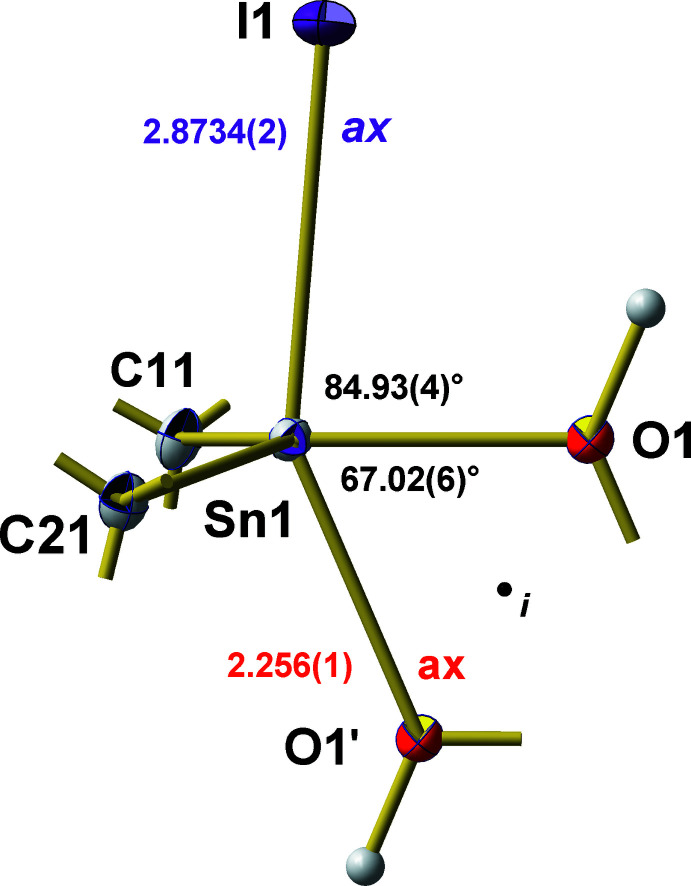
Ball-and-stick model of the trigonal–bipyramidal coordination environment of the tin atom in the dimeric mol­ecule of ^*t*^Bu_2_Sn(OH)I with bond lengths (Å) and angles (°) characterizing the polyhedron axes. For clarity, methyl groups of the ^*t*^Bu ligands are stripped down to the carbon–carbon bonds drawn as shortened sticks. Atom O1′ is generated by symmetry code −*x*, −*y* + 1, −*z* + 1.

Within the trigonal–bipyramidal coordination of the Sn^IV^ atom (Fig. 2 ▸), both *tert*-butyl groups are in equatorial (eq) positions in correspondence with the predictions of the VSEPR concept. The bond angle enclosed by the two *tert*-butyl groups of 126.81 (8)° is identical with the value [126.89 (9)°] in the co-crystal and lies in the range 122.0 (2) to 129.3 (1)° of C—Sn—C angles found in the other *hydroxide-halide*s. The iodine atom adopts an axial (ax) position and one of the bridging hydroxyl groups is in an equatorial, the other in an axial position. As a result of dimerization *via* the hydroxyl groups, the axis of the trigonal bipyramid strongly deviates from linearity [I_ax_—Sn—(OH)_ax_ = 151.94 (4)°]. In addition, the Sn—I distance of 2.8734 (2) Å is only marginally shorter than in the co-crystal [Sn—I = 2.8852 (2) Å], both being significantly longer than the sum (2.78 Å) of the covalent radii (Cordero *et al.*, 2008 ▸) of tin (1.39 Å) and iodine (1.39 Å) and much longer than the mean Sn—I distance of 2.661 (2) (Reuter & Pawlak, 2001 ▸) in tin(IV) iodide, SnI_4_, with tetra­hedrally coordinated tin.

**Figure 3:**
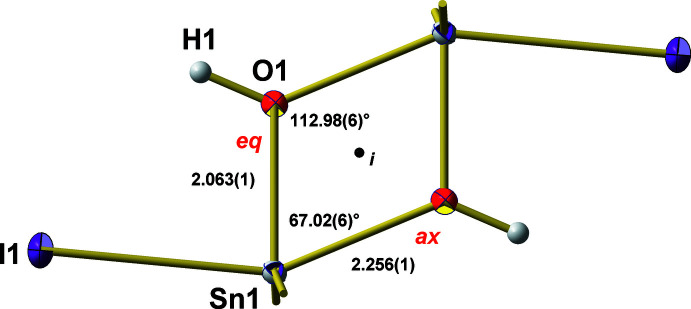
Ball-and-stick model of the four-membered, centrosymmetric {Sn—O}_2_ ring in the dimeric mol­ecule of ^*t*^Bu_2_Sn(OH)I with bond lengths (Å) and angles (°) underlining its rhombic shape as the result of axially (ax) and equatorially (eq) bonded O atoms.

Because of the centrosymmetric nature of the dimer, the central four-membered {Sn—O}_2_ ring is exactly planar. Its rhombic shape (Fig. 3 ▸) is characterized by acute angles [67.02 (6)°] at tin and obtuse ones [112.98 (6)°] at oxygen. Moreover, these rings exhibit two different Sn—O distances depending on the position (ax/eq) of the oxygen atom in the trigonal–bipyramidal coordination environment of tin(IV): Sn—(OH)_ax_ = 2.256 (1) Å *versus* Sn—(OH)_eq_ = 2.063 (1) Å. All these structural features are typical. For example, for the other four-membered {Sn—O}_2_ rings of *hydroxide halides* with *R* = ^*t*^Bu, *Hal* = F, Cl, Br, the Sn—O—Sn angles range from 109.9 (2) to 112.5 (3)°, the O—Sn—O angles from 67.9 (3) to 70.1 (2)°, the Sn—(OH)_eq_ distances from 2.012 (5) to 2.048 (10) Å, and the Sn—(OH)_ax_ distances from 2.199 (5) to 2.25.7 (16) Å (Puff *et al.*, 1985 ▸) *.*

## 3. Supra­molecular features

**Figure 4:**
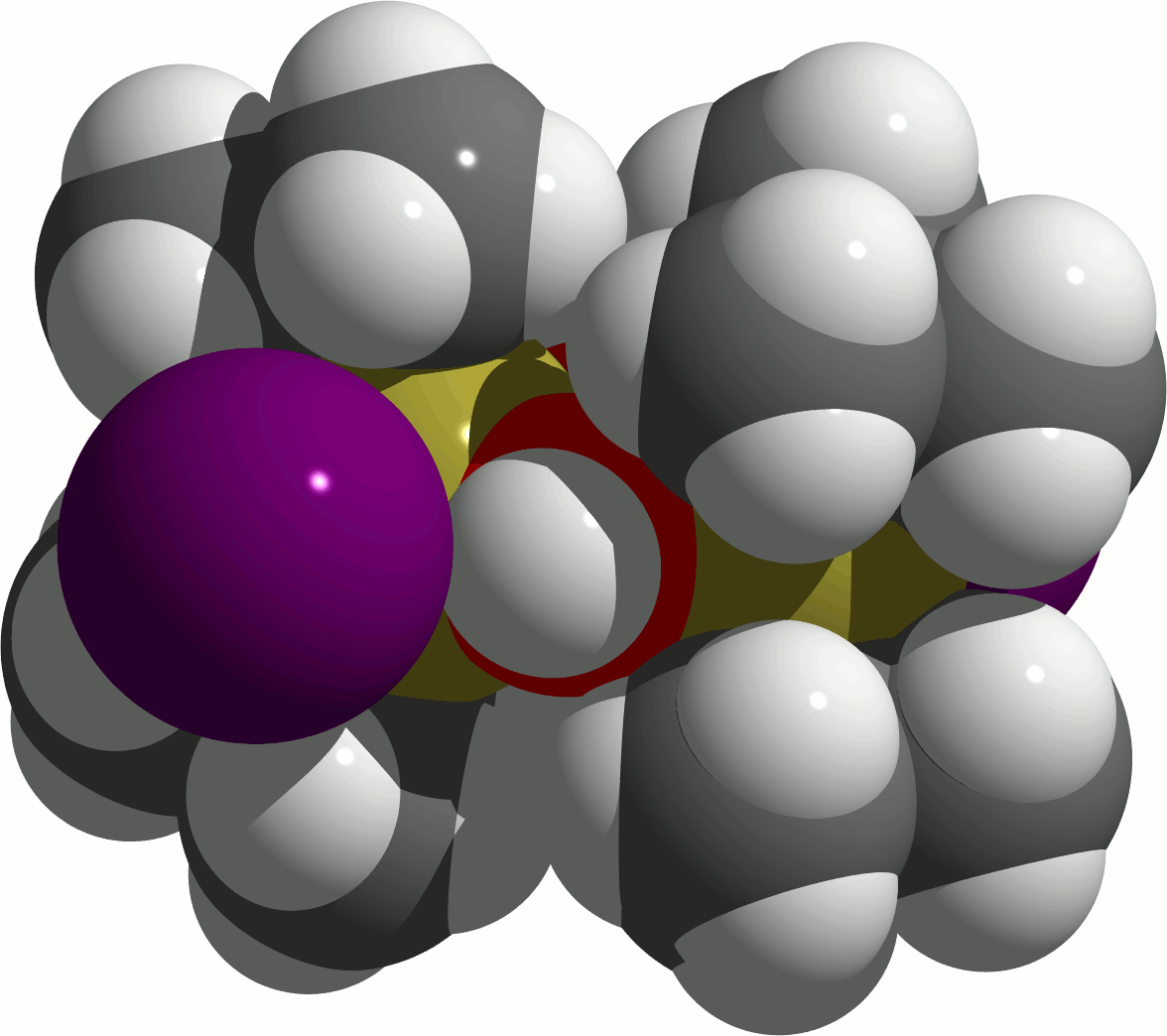
Space-filling model of the dimeric mol­ecule of ^*t*^Bu_2_Sn(OH)I showing the OH group wedged in between the iodine atom and the *tert*-butyl groups. Colour code: I = violet, H = white, C = grey, O = red, Sn = brass-coloured.

**Figure 5:**
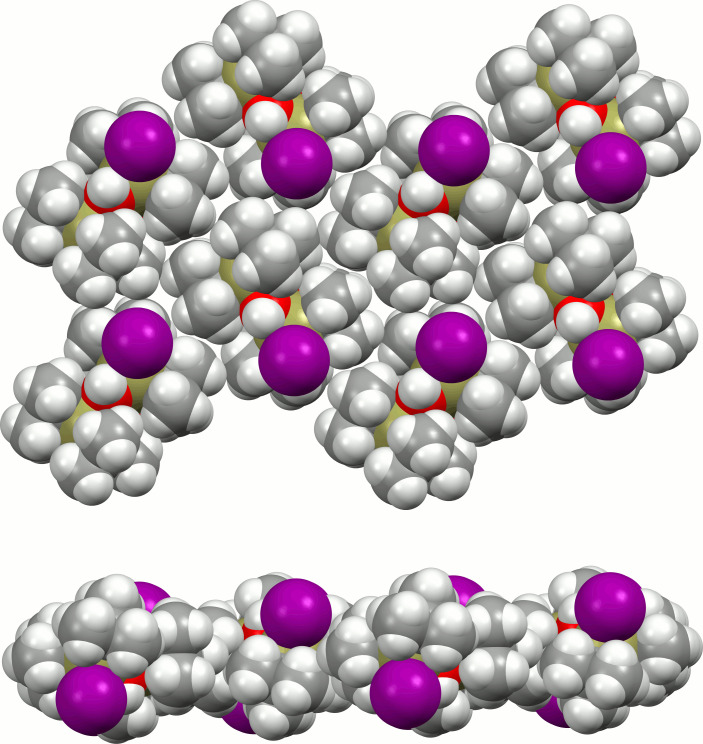
Space-filling model showing the layer-like arrangement of the dimeric [^*t*^BuSn(OH)I]_2_ mol­ecules in the crystal structure. Top: top view; bottom: side view; colour code as in Fig. 4 ▸.

**Figure 6:**
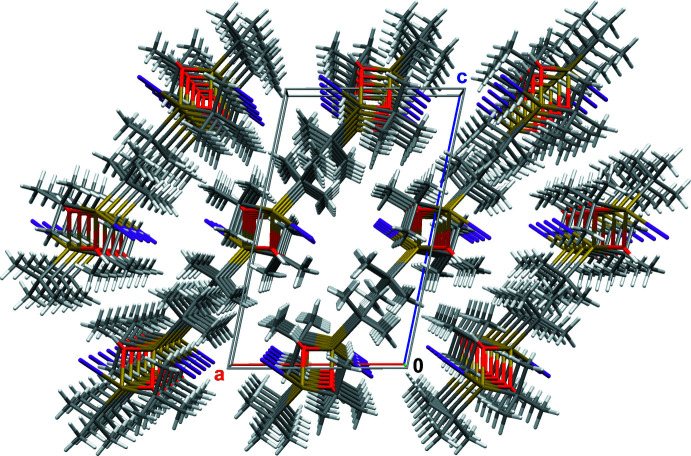
Stick-model showing the arrangement of layers with respect to the unit cell; colour code as in Fig. 4 ▸.

While the hydroxyl groups of the [^*t*^Bu_2_Sn(OH)I]_2_ mol­ecules of the co-crystallizate (Reuter & Wilberts, 2014 ▸) are involved in OH⋯O hydrogen-bonding to DMSO mol­ecules, those of all other [^*t*^Bu_2_Sn(OH)*Hal*]_2_ mol­ecules develop inter­molecular O—H⋯*Hal* bonds resulting in a chain-like arrangement of the corresponding mol­ecules in the crystal. In contrast, there are no hydrogen bonds in the crystal structure of the title compound as the voluminous iodine atoms (Fig. 4 ▸) prevent significant inter­molecular OH⋯I inter­actions (Table 1 ▸). Hence, only van der Waals forces exist between the individual mol­ecules, resulting in a layer-like arrangement (Fig. 5 ▸) with the Sn—I bonds perpendicular to the layer plane. These layers expand perpendicular to the [101] direction (Fig. 6 ▸).

## 4. Synthesis and crystallization

Yellow block-like single crystals of the title compound were obtained after several years of storage in a sample of (^*t*^Bu_2_Sn)_2_I_2_ originally prepared by the reaction of the cyclo­tetra­stannane (^*t*^Bu_2_Sn)_4_ with I_2_ in toluene at elevated temperature in a molar ratio of 1:2. As other molar ratios and temperatures result in the formation of (^*t*^Bu_2_Sn)_4_I_2_ or ^*t*^Bu_2_SnI_2_ (Farrar & Skinner, 1964 ▸; Adams & Dräger, 1985 ▸; Puff *et al.*, 1989 ▸), it seems possible that the sample was contaminated with the latter one, which reacts over the long time of storage with atmospheric moisture to give the title compound.

## 5. Refinement

Crystal data, data collection and structure refinement details are summarized in Table 2 ▸. All hydrogen atoms were clearly identified in difference-Fourier syntheses. Those of the *tert*-butyl groups were refined with calculated positions (C—H = 0.98 Å) and a common *U*_iso_(H) parameter for each of the methyl groups. The position of the H atom of the OH group was refined with a fixed O—H distance of 0.96 Å and the *U*_iso_(H) parameter refined freely.

## Supplementary Material

Crystal structure: contains datablock(s) I. DOI: 10.1107/S205698902200514X/wm5646sup1.cif

Structure factors: contains datablock(s) I. DOI: 10.1107/S205698902200514X/wm5646Isup2.hkl

CCDC reference: 2172420

Additional supporting information:  crystallographic information; 3D view; checkCIF report

## Figures and Tables

**Table 1:** Hydrogen-bond geometry (Å, °)

*D*—H⋯*A*	*D*—H	H⋯*A*	*D*⋯*A*	*D*—H⋯*A*
O1—H1⋯I1	0.96	2.93	3.3862 (14)	111

**Table 2:** Experimental details

Crystal data	
Chemical formula	[Sn(C_4_H_9_)_2_I(OH)]
*M*_r_	376.82
Crystal system, space group	Monoclinic, *P*2_1_/*n*
Temperature (K)	100
*a*, *b*, *c* (Å)	8.4903 (4), 10.8848 (5), 13.5107 (6)
β (°)	101.881 (2)
*V* (Å^3^)	1221.85 (10)
*Z*	4
Radiation type	Mo *K*α
μ (mm^−1^)	4.58
Crystal size (mm)	0.24 × 0.12 × 0.09

Data collection	
Diffractometer	Bruker APEXII CCD
Absorption correction	Multi-scan (*SADABS*; Krause *et al.*, 2015 ▸)
*T*_min_, *T*_max_	0.457, 0.715
No. of measured, independent and observed [*I* > 2σ(*I*)] reflections	48755, 2950, 2761
*R*_int_	0.064
(sin θ/λ)_max_ (Å^−1^)	0.661

Refinement	
*R*[*F* ^2^ > 2σ(*F* ^2^)], *wR*(*F* ^2^), *S*	0.016, 0.037, 1.08
No. of reflections	2950
No. of parameters	114
H-atom treatment	H-atom parameters constrained
Δρ_max_, Δρ_min_ (e Å^−3^)	0.85, −0.49

Computer programs: *APEX2* and *SAINT* (Bruker, 2009 ▸), *SHELXS* (Sheldrick, 2008 ▸), *SHELXL* (Sheldrick, 2015 ▸), *DIAMOND* (Brandenburg, 2006 ▸), *Mercury* (Macrae *et al.*, 2020 ▸) and *publCIF* (Westrip, 2010 ▸).

## supplementary crystallographic information

### Crystal data

**Table:**

[Sn(C_4_H_9_)_2_I(OH)]	*F*(000) = 712
*M**_r_* = 376.82	*D*_x_ = 2.048 Mg m^−^^3^
Monoclinic, *P*2_1_/*n*	Mo *K*α radiation, λ = 0.71073 Å
*a* = 8.4903 (4) Å	Cell parameters from 9771 reflections
*b* = 10.8848 (5) Å	θ = 2.4–29.2°
*c* = 13.5107 (6) Å	µ = 4.58 mm^−^^1^
β = 101.881 (2)°	*T* = 100 K
*V* = 1221.85 (10) Å^3^	Block, yellow
*Z* = 4	0.24 × 0.12 × 0.09 mm

### Data collection

**Table:**

Bruker APEXII CCD diffractometer	2761 reflections with *I* > 2σ(*I*)
φ and ω scans	*R*_int_ = 0.064
Absorption correction: multi-scan (*SADABS*; Krause *et al.*, 2015)	θ_max_ = 28.0°, θ_min_ = 2.6°
*T*_min_ = 0.457, *T*_max_ = 0.715	*h* = −11→11
48755 measured reflections	*k* = −14→13
2950 independent reflections	*l* = −17→17

### Refinement

**Table:**

Refinement on *F*^2^	Hydrogen site location: mixed
Least-squares matrix: full	H-atom parameters constrained
*R*[*F*^2^ > 2σ(*F*^2^)] = 0.016	*w* = 1/[σ^2^(*F*_o_^2^) + (0.0075*P*)^2^ + 1.3137*P*] where *P* = (*F*_o_^2^ + 2*F*_c_^2^)/3
*wR*(*F*^2^) = 0.037	(Δ/σ)_max_ = 0.003
*S* = 1.08	Δρ_max_ = 0.85 e Å^−^^3^
2950 reflections	Δρ_min_ = −0.49 e Å^−^^3^
114 parameters	Extinction correction: SHELXL-2014/7 (Sheldrick 2015, Fc^*^=kFc[1+0.001xFc^2^λ^3^/sin(2θ)]^-1/4^
0 restraints	Extinction coefficient: 0.00139 (11)
Primary atom site location: structure-invariant direct methods	

### Special details

**Table:**

Geometry. All esds (except the esd in the dihedral angle between two l.s. planes) are estimated using the full covariance matrix. The cell esds are taken into account individually in the estimation of esds in distances, angles and torsion angles; correlations between esds in cell parameters are only used when they are defined by crystal symmetry. An approximate (isotropic) treatment of cell esds is used for estimating esds involving l.s. planes.

### Fractional atomic coordinates and isotropic or equivalent isotropic displacement parameters (Å^2^)

**Table:**

	*x*	*y*	*z*	*U*_iso_*/*U*_eq_	
I1	0.31459 (2)	0.21150 (2)	0.53430 (2)	0.01973 (5)	
Sn1	0.06240 (2)	0.37120 (2)	0.43364 (2)	0.01006 (5)	
C21	0.1879 (2)	0.44568 (19)	0.32082 (15)	0.0137 (4)	
C22	0.2375 (3)	0.3375 (2)	0.26155 (17)	0.0193 (5)	
H22A	0.1409	0.2956	0.2247	0.032 (4)*	
H22B	0.3027	0.2798	0.3086	0.032 (4)*	
H22C	0.3004	0.3679	0.2135	0.032 (4)*	
C23	0.3377 (3)	0.5141 (2)	0.37485 (17)	0.0190 (4)	
H23A	0.3972	0.5445	0.3249	0.023 (4)*	
H23B	0.4064	0.4582	0.4218	0.023 (4)*	
H23C	0.3059	0.5835	0.4126	0.023 (4)*	
C24	0.0749 (3)	0.5304 (2)	0.24902 (16)	0.0190 (4)	
H24A	0.1273	0.5569	0.1944	0.027 (4)*	
H24B	0.0495	0.6025	0.2863	0.027 (4)*	
H24C	−0.0246	0.4863	0.2203	0.027 (4)*	
C11	−0.1328 (3)	0.23567 (19)	0.40781 (16)	0.0153 (4)	
C12	−0.2895 (3)	0.2973 (2)	0.35517 (19)	0.0233 (5)	
H12A	−0.2754	0.3331	0.2910	0.029 (4)*	
H12B	−0.3175	0.3623	0.3987	0.029 (4)*	
H12C	−0.3759	0.2362	0.3421	0.029 (4)*	
C13	−0.0867 (3)	0.1346 (2)	0.34001 (18)	0.0214 (5)	
H13A	−0.0798	0.1697	0.2742	0.028 (4)*	
H13B	−0.1687	0.0699	0.3304	0.028 (4)*	
H13C	0.0178	0.0997	0.3721	0.028 (4)*	
C14	−0.1500 (3)	0.1809 (2)	0.50925 (18)	0.0236 (5)	
H14A	−0.2351	0.1185	0.4979	0.029 (4)*	
H14B	−0.1781	0.2461	0.5526	0.029 (4)*	
H14C	−0.0480	0.1431	0.5423	0.029 (4)*	
O1	0.09927 (17)	0.46366 (13)	0.56977 (10)	0.0150 (3)	
H1	0.1841	0.4272	0.6190	0.070 (12)*	

### Atomic displacement parameters (Å^2^)

**Table:**

	*U*^11^	*U*^22^	*U*^33^	*U*^12^	*U*^13^	*U*^23^
I1	0.02092 (9)	0.01756 (8)	0.01844 (8)	0.00750 (5)	−0.00125 (6)	0.00161 (5)
Sn1	0.01100 (8)	0.00881 (8)	0.01046 (7)	0.00010 (5)	0.00239 (5)	−0.00050 (5)
C21	0.0164 (10)	0.0130 (10)	0.0128 (9)	−0.0014 (8)	0.0051 (8)	−0.0012 (8)
C22	0.0235 (12)	0.0175 (11)	0.0188 (11)	−0.0001 (9)	0.0085 (9)	−0.0039 (9)
C23	0.0180 (11)	0.0196 (11)	0.0206 (11)	−0.0048 (9)	0.0067 (9)	−0.0020 (9)
C24	0.0236 (11)	0.0176 (11)	0.0158 (10)	0.0020 (9)	0.0040 (9)	0.0041 (8)
C11	0.0170 (10)	0.0131 (10)	0.0171 (10)	−0.0037 (8)	0.0064 (8)	−0.0040 (8)
C12	0.0153 (11)	0.0226 (12)	0.0308 (13)	−0.0029 (9)	0.0018 (9)	−0.0057 (10)
C13	0.0223 (12)	0.0173 (11)	0.0255 (12)	−0.0036 (9)	0.0070 (9)	−0.0083 (9)
C14	0.0295 (13)	0.0198 (12)	0.0250 (12)	−0.0054 (10)	0.0135 (10)	0.0012 (9)
O1	0.0166 (7)	0.0147 (7)	0.0126 (7)	0.0047 (6)	0.0004 (6)	−0.0021 (6)

### Geometric parameters (Å, º)

**Table:**

I1—Sn1	2.8734 (2)	C24—H24C	0.9800
Sn1—O1	2.0631 (1)	C11—C12	1.528 (3)
Sn1—C21	2.187 (2)	C11—C14	1.529 (3)
Sn1—C11	2.193 (2)	C11—C13	1.533 (3)
Sn1—O1^i^	2.2564 (1)	C12—H12A	0.9800
C21—C23	1.524 (3)	C12—H12B	0.9800
C21—C24	1.526 (3)	C12—H12C	0.9800
C21—C22	1.531 (3)	C13—H13A	0.9800
C22—H22A	0.9800	C13—H13B	0.9800
C22—H22B	0.9800	C13—H13C	0.9800
C22—H22C	0.9800	C14—H14A	0.9800
C23—H23A	0.9800	C14—H14B	0.9800
C23—H23B	0.9800	C14—H14C	0.9800
C23—H23C	0.9800	O1—Sn1^i^	2.2563 (14)
C24—H24A	0.9800	O1—H1	0.9600
C24—H24B	0.9800		
			
O1—Sn1—C21	115.73 (7)	C21—C24—H24C	109.5
O1—Sn1—C11	116.17 (7)	H24A—C24—H24C	109.5
C21—Sn1—C11	126.81 (8)	H24B—C24—H24C	109.5
O1—Sn1—O1^i^	67.02 (6)	C12—C11—C14	110.71 (19)
C21—Sn1—O1^i^	94.13 (7)	C12—C11—C13	109.99 (18)
C11—Sn1—O1^i^	95.51 (7)	C14—C11—C13	109.84 (19)
O1—Sn1—I1	84.93 (4)	C12—C11—Sn1	109.60 (14)
C21—Sn1—I1	97.57 (5)	C14—C11—Sn1	109.16 (14)
C11—Sn1—I1	97.67 (6)	C13—C11—Sn1	107.47 (14)
O1^i^—Sn1—I1	151.94 (4)	C11—C12—H12A	109.5
C23—C21—C24	111.25 (18)	C11—C12—H12B	109.5
C23—C21—C22	109.50 (17)	H12A—C12—H12B	109.5
C24—C21—C22	109.69 (17)	C11—C12—H12C	109.5
C23—C21—Sn1	109.00 (13)	H12A—C12—H12C	109.5
C24—C21—Sn1	109.55 (13)	H12B—C12—H12C	109.5
C22—C21—Sn1	107.78 (14)	C11—C13—H13A	109.5
C21—C22—H22A	109.5	C11—C13—H13B	109.5
C21—C22—H22B	109.5	H13A—C13—H13B	109.5
H22A—C22—H22B	109.5	C11—C13—H13C	109.5
C21—C22—H22C	109.5	H13A—C13—H13C	109.5
H22A—C22—H22C	109.5	H13B—C13—H13C	109.5
H22B—C22—H22C	109.5	C11—C14—H14A	109.5
C21—C23—H23A	109.5	C11—C14—H14B	109.5
C21—C23—H23B	109.5	H14A—C14—H14B	109.5
H23A—C23—H23B	109.5	C11—C14—H14C	109.5
C21—C23—H23C	109.5	H14A—C14—H14C	109.5
H23A—C23—H23C	109.5	H14B—C14—H14C	109.5
H23B—C23—H23C	109.5	Sn1—O1—Sn1^i^	112.98 (6)
C21—C24—H24A	109.5	Sn1—O1—H1	111.7
C21—C24—H24B	109.5	Sn1^i^—O1—H1	135.3
H24A—C24—H24B	109.5		

Symmetry code: (i) −*x*, −*y*+1, −*z*+1.

### Hydrogen-bond geometry (Å, º)

**Table:**

*D*—H···*A*	*D*—H	H···*A*	*D*···*A*	*D*—H···*A*
O1—H1···I1	0.96	2.93	3.3862 (14)	111
